# The Book World of Medicine and Science

**Published:** 1898-08-27

**Authors:** 


### The Book World of Medicine and Science.

Twenty-two Years' Experience in the Treatment of Cancerous and Other Tumours. By Herbert Snow, M.D. (London: Baillière, Tindall, and Cox. 1898.)

Were it not that this book deals with a subject that is of the gravest import, one would be almost inclined to think that the writer intended that it should not be taken seriously as the outcome of twenty-two years' experience, but rather as a rough sketch of some of his views on the subject of "Cancer" in so far as they are at variance with those which are more usually accepted. The book opens with a discussion on the increase of malignant disease, of which there must be some doubt, since the writer does not clearly define what he would include under the terms that he uses. Among the measures proposed for the more satisfactory treatment of cancer is that the medical education of the country should be altered at its source, and that first and foremost there should be a "renovation of that peculiar mediæval anachronism, the College of Surgeons." He would have us further to believe that it is an essential that there should be a drastic change in the general hospitals of the land, especially those with medical schools, apparently on the lines that no case that can be considered a "special" one (and what case is not?) is to be treated at such, but should be at once sent to a "special" hospital, and there treated, so that there should be no chance that any medical student should be contaminated by what is the wrong treatment in vogue at his school. Under the heading of "Causation," the author states that worry, trouble, and anxiety, the products of civilisation, are the chief exciters of "cancer," but as, immediately opposite, the statement is to be found that pampered dogs are nearly the only members of the animal kingdom, besides men, who are subject to "cancer," one is led to wonder whether such dogs are also the victims of worry and trouble! Again, such statements as that uncivilised nations are very free from malignant disorders must be taken with some reserve, since our knowledge of their diseases is none too great. The microscopic anatomy of the various tumours under consideration is, to say the least, very imperfect, and we fail to see what good can accrue from introducing such into a work of this sort. As an example of this imperfection we may instance the account given of so-called "rodent ulcer." Here we are told that it mimics hair-follicle structures—hardly an accurate statement of what is the typical appearance of a section of a rodent growth. No mention is made of the possible occurrence of epithelial pearls, nor of the different forms of the growth. Further, it is asserted that the distinction between this affection and epithelioma is of no practical importance, a statement with which we cannot agree. When treatment is dealt with, the author gives it as his opinion that the medicinal use of opium should be commenced in any case where it is thought that the "cancer" is incurable. While some will agree with this, it seems quite doubtful as yet whether experience will bear out the belief of the writer that opium will tend to diminish the rapidity of the growth, and to prevent its so-called recurrence after operation. With regard to the operative treatment of malignant disease, Dr. Snow does not seem to possess any highly optimistic views. He says of mammary "cancer" that unless it be attacked within the first six weeks of its appearance, it is almost hopeless to eradicate it; but, unfortunately for prognosis, it is not easy to say when the first deposit of the insidious disease took place. There is no doubt that the work before us contains many details as to the subject of which it treats which are of much interest, but these appear to be thrown together in some confusion, and we may fairly doubt whether the work will add much to our knowledge of "cancerous" and other tumours.

Twentieth Century Practice of Medicine. Edited by Thomas L. Stedman, M.D. Vol. XI.—Diseases of the Nervous System. (London: 1897. Sampson Low, Marston, and Co. Pp. 962, with illustrations.)

This volume completes the account of the nervous system in the series. Half of it is occupied by Dr. James Hendrie Lloyd's account of the diseases of the cerebro-spinal and sympathetic nerves. The value of his physiological introduction to this subject is considerably impaired by the fact that he uses the word "electrotonus" in an inaccurate sense throughout. Of the other introductory sections, that on the disorders of nutrition is the best. The diseases of the individual nerves are well sketched; but this part of the book, valuable as it is, suffers by the inevitable comparison with Bernhardt's masterly work which appeared almost contemporaneously. Of the cranial nerves, the second is most fully discussed, but the account of albuminuric retinitis lacks definiteness. In other parts too much space is taken up with trivialities such as a discussion of the relations of the sense of smell to the pigment cells in the olfactory epithelium. Per contra, it is curious to note that no mention is made of the association and pathological affinity of ophthalmoplegia with progressive muscular atrophy. Some of the diseases of special nerves are, however, well and accurately described, particularly tic-douloureux, facial palsy, and affections of the glosso-pharyngeal. Dr. Lloyd is well versed in the results of recent anatomical investigations and their application to diagnosis. His account of multiple neuritis is somewhat diffuse and lacking in orderly arrangement, while the attempt to speak of it as a single disease sometimes results in want of accuracy. Thus we read (p. 416), "Sensation is usually profoundly affected in every case of multiple neuritis"; the inapplicability of this statement to lead neuritis is obvious. Dr. C. K. Mills writes on trophoneuroses, of which the first is called hemifacial atrophy, a barbarism for which it is difficult to find a justification. But the account of this and other hypertrophic and atrophic curiosities is interesting, and the same may be said of the rather brief descriptions of Raynaud's disease and perforating ulcer of the foot; that of ainhum is condensed from the well-known paper of Dr. Pyle. The other trophoneuroses, sclerodema, acromegaly, and adiposis dolorosa are described by a third distinguished member of the Philadelphia school, Dr. Dercum. This section is admirably done, the chapter on Sclerodema being certainly one of the best that have ever appeared on the subject. The general subject of diseases of the spinal cord is handled dictionary-wise by Dr. Brus, of Hanover, and Dr. Windschied, of Leipsic. No account of the anatomy or physiology of the cord is given, and the article is in consequence disconnected and a little unconvincing; the chapter on myelitis is, however, very thorough and accurate, and the etiology of infantile anterior poliomyelitis is well discussed from the modern standpoint. Dr. Möbius' article on tabes is by far the best thing in the book, and is indeed a model of close and accurate writing, sound judgment, and apt illustration, but Professor Strümpell's account of the combined system diseases of the cord is quite unworthy of his reputation. The book concludes with an article on the psychology of pain, by Dr. Witmer, of Philadelphia, which is of great analytical interest, although its appearance in a professedly practical work seems a little incongruous. On the whole the volume is unequal, and a little disappointing after the exceptionally high standard reached by its predecessors. But one or two of the essays are almost brilliant enough to redeem the bulk of the work.

